# Association of Hepatic Steatosis Burden With Microalbuminuria in Patients With Type 2 Diabetes Mellitus: A Cross-Sectional Study

**DOI:** 10.7759/cureus.110015

**Published:** 2026-05-31

**Authors:** Komal S Jog, Harini Bopaiah, Guru Yogendra Muthyal, Sai Deepika R

**Affiliations:** 1 Department of Endocrinology, Sri Devaraj Urs Medical College, Kolar, IND; 2 Department of Radiodiagnosis, Sri Devaraj Urs Medical College, Kolar, IND; 3 Department of Biochemistry, Sri Devaraj Urs Medical College, Kolar, IND

**Keywords:** diabetic kidney disease, hepatic steatosis, masld, microalbuminuria, urinary-albumin-creatinine-ratio (uacr)

## Abstract

Introduction

Metabolic dysfunction-associated steatotic liver disease (MASLD) is increasingly recognized as a multisystem disorder associated with cardiometabolic complications. Emerging evidence suggests a potential link between hepatic steatosis and diabetic kidney disease, particularly microalbuminuria. This study aimed to evaluate the association between hepatic steatosis burden and urinary albumin-creatinine ratio (UACR) in patients with type 2 diabetes mellitus.

Methods

In this cross-sectional study, 95 patients with type 2 diabetes mellitus were evaluated. Hepatic steatosis was assessed using computed tomography-derived liver attenuation index (LAI) and steatosis grading. UACR was used as a marker of microalbuminuria. Correlation analysis and multivariate linear regression were performed to determine the relationship between hepatic steatosis parameters and albuminuria.

Results

The mean age of participants was 53.16 ± 9.94 years, with a mean BMI of 24.97 ± 2.03 kg/m². UACR increased progressively with higher grades of hepatic steatosis (Kruskal-Wallis H = 49.53, p < 0.001). LAI showed a significant negative correlation with UACR (r = −0.66, p < 0.001). In multivariate regression analysis, LAI (β = −1.69, p < 0.001) and BMI (β = 2.13, p = 0.004) were independently associated with UACR.

Conclusion

Hepatic steatosis burden is significantly associated with microalbuminuria in patients with type 2 diabetes mellitus, suggesting its potential role as an early marker of renal endothelial dysfunction.

## Introduction

Type 2 diabetes mellitus (T2DM) is a major global health problem and is frequently associated with metabolic comorbidities that contribute to increased cardiovascular and renal morbidity. Microalbuminuria, assessed by the urine albumin-creatinine ratio (UACR), is an early marker of diabetic nephropathy and generalized endothelial dysfunction and predicts progression to chronic kidney disease [1].

Metabolic dysfunction-associated steatotic liver disease (MASLD), previously termed non-alcoholic fatty liver disease (NAFLD), is increasingly recognized as the hepatic manifestation of metabolic syndrome and is highly prevalent among individuals with T2DM, with more than 60% showing evidence of hepatic steatosis [2]. MASLD is closely linked to systemic metabolic dysfunction, including insulin resistance, dyslipidemia, chronic low-grade inflammation, and endothelial dysfunction [3].

Accumulating evidence suggests a relationship between hepatic steatosis and diabetic kidney disease [4-8]. Proposed mechanisms include systemic insulin resistance, oxidative stress, inflammatory cytokine release, and activation of the renin-angiotensin system, contributing to glomerular injury and albuminuria [5].

Imaging-based indices such as the Liver Attenuation Index (LAI) on computed tomography (CT) provide non-invasive quantitative estimates of hepatic fat content. However, the association between hepatic steatosis burden measured by the LAI and early renal injury markers such as UACR remains inadequately explored in T2DM. Therefore, this study aimed to evaluate the correlation between microalbuminuria and hepatic steatosis burden quantified using CT-based indices (LAI) in patients with T2DM.

## Materials and methods

This was a cross-sectional observational study conducted over five months in the Department of Endocrinology at the RL Jalappa Hospital, Kolar, Karnataka, India, attached to the Sri Devraj Urs Academy of Higher Education and Research, from December 2025 to April 2026. The study was approved by the Central Ethics Committee, Sri Devraj Urs Academy of Higher Education and Research (reference number: SDUAHER/R&D/CEC/SDUMC-F/65/NF/-2025-26), and written informed consent was obtained from all participants.

Study population

Adult patients (aged ≥18 years) diagnosed with T2DM according to the American Diabetes Association (AMA) attending the outpatient and inpatient departments during the study period were recruited for the study. Patients with a history of significant alcohol consumption, known chronic liver disease of other etiologies, including viral hepatitis, autoimmune liver disease, or drug-induced liver disease, overt proteinuria, established chronic kidney disease, or acute illness/infection at the time of evaluation were excluded from the study.

Sample Size

Based on prior literature reporting a correlation of r = 0.331 [4] between steatosis and UACR, a sample size of 64 achieves 80% power to detect this correlation (α = 0.05); allowing 10% attrition increases the recruitment target to 72. For 90% power, the minimum is 86 (≈96 with 10% attrition). We therefore planned to recruit 80-100 participants to ensure adequate power and robustness to missing data.

Clinical and biochemical assessment

Demographic data, including age and sex, were recorded. Anthropometric measurements such as BMI were obtained using standard methods. Blood samples were collected after an overnight fast for estimation of fasting plasma glucose, glycated hemoglobin (HbA1c), and lipid profile including total cholesterol, triglycerides, high-density lipoprotein cholesterol (HDL-C), and low-density lipoprotein cholesterol (LDL-C). UACR was measured in a spot urine sample on a single occasion. Microalbuminuria was defined as a UACR of 30-300 mg/g creatinine, while macroalbuminuria was defined as a UACR >300 mg/g creatinine. UACR was estimated in a fully automated Vitros 5600 analyser (QuidelOrtho Corporation, San Diego, California, United States).

Assessment of hepatic steatosis

Hepatic steatosis burden was assessed qualitatively and graded into grades 1-3 using two-dimensional ultrasonography with Philips EPIQ 5/Philips Affiniti 70 ultrasonography systems (Koninklijke Philips N.V., Amsterdam, Netherlands) and a curvilinear probe. 

Quantitative assessment was performed using CT-based evaluation of the LAI and L/S attenuation ratio. The L/S ratio was calculated by measuring the attenuation values of the liver and spleen, and the ratio was derived by dividing the mean hepatic attenuation by the splenic attenuation. Lower L/S ratios indicate greater hepatic fat accumulation and higher hepatic steatosis burden. 

All CT measurements were performed on unenhanced (non-contrast) CT images. No contrast-enhanced phase images were used, as contrast administration alters hepatic attenuation values and would confound fat quantification. Three circular regions of interest (ROIs), each approximately 1-2 cm², were placed in the right hepatic lobe: one each in segments V, VI, and VII. ROIs were carefully positioned to avoid hepatic vessels, biliary structures, focal fatty infiltration, and subcapsular regions. Mean attenuation was recorded in Hounsfield units (HU) for each ROI, and the average of the three values was used as the representative hepatic attenuation. Three similarly sized ROIs were placed in the splenic parenchyma, avoiding splenic vessels and capsular margins. The mean of the three measurements was used as representative splenic attenuation.

LAI (HU) = Mean liver attenuation (HU) minus Mean spleen attenuation (HU). An LAI below -10 HU was used as the CT threshold for hepatic steatosis [9].

CT measurements were independently performed by two radiologists from the Department of Radiodiagnosis. Each radiologist assessed all CT studies independently and was blinded to all clinical and biochemical data at the time of analysis.

Statistical analysis

Data were analyzed using IBM SPSS Statistics for Windows, version 22.0 (IBM Corp., Armonk, New York, United States). Continuous variables were expressed as mean ± standard deviation (SD) or median (interquartile range (IQR)) based on distribution, while categorical variables were presented as frequencies and percentages. Normality was assessed using the Shapiro-Wilk test. Group comparisons were performed using Student’s t-test or one-way analysis of variance (ANOVA) for normally distributed variables and the Kruskal-Wallis test for non-parametric data. Correlation between UACR and hepatic steatosis parameters, including LAI, was evaluated using Pearson or Spearman correlation analysis. Multivariate linear regression analysis was performed to identify independent determinants of UACR. A two-tailed p-value <0.05 was considered statistically significant.

## Results

Baseline characteristics

A total of 95 patients with T2DM were included in the study. The mean age of the study population was 53.16 ± 9.94 years (range: 27-71 years). The cohort comprised 49 male (51.6%) and 46 female (48.4%) patients. The mean BMI was 24.97 ± 2.03 kg/m² (range: 20.49-29.24 kg/m²).

With respect to renal parameters, the mean serum creatinine level was 0.81 ± 0.35 mg/dL (range: 0.40-2.00 mg/dL), while the mean estimated glomerular filtration rate (eGFR) calculated using the CKD-EPI equation was 95.91 ± 25.45 mL/min/1.73 m² (range: 33.36-142.74 mL/min/1.73 m²). The mean UACR was 36.14 ± 17.47 mg/g, with values ranging from 4.97 to 86.63 mg/g.

Evaluation of hepatic steatosis showed that the majority of patients had grade 1 steatosis (n = 62, 65.3%), followed by grade 2 steatosis in 19 patients (20.0%) and grade 3 steatosis in 12 patients (12.6%), while two participants (2.1%) had no evidence of steatosis (grade 0). The mean LAI among the study participants was 36.16 ± 5.82.

The baseline characteristics of the study population are summarized in Table 1.

**Table 1:** Baseline characteristics of the study population (N = 95) BMI, body mass index; HbA1c, glycated hemoglobin; UACR, urinary albumin-to-creatinine ratio

Variable	Value
Age (years), mean ± SD	53.16 ± 9.94
Male, n (%)	49 (51.6%)
Female, n (%)	46 (48.4%)
Duration of diabetes (years), mean ± SD	7.78 ± 7.39
BMI (kg/m²), mean ± SD	24.97 ± 2.03
HbA1c (%), mean ± SD	9.65 ± 2.31
UACR (mg/g), mean ± SD	36.14 ± 17.

Association between steatosis grade and UACR

**Figure 1:**
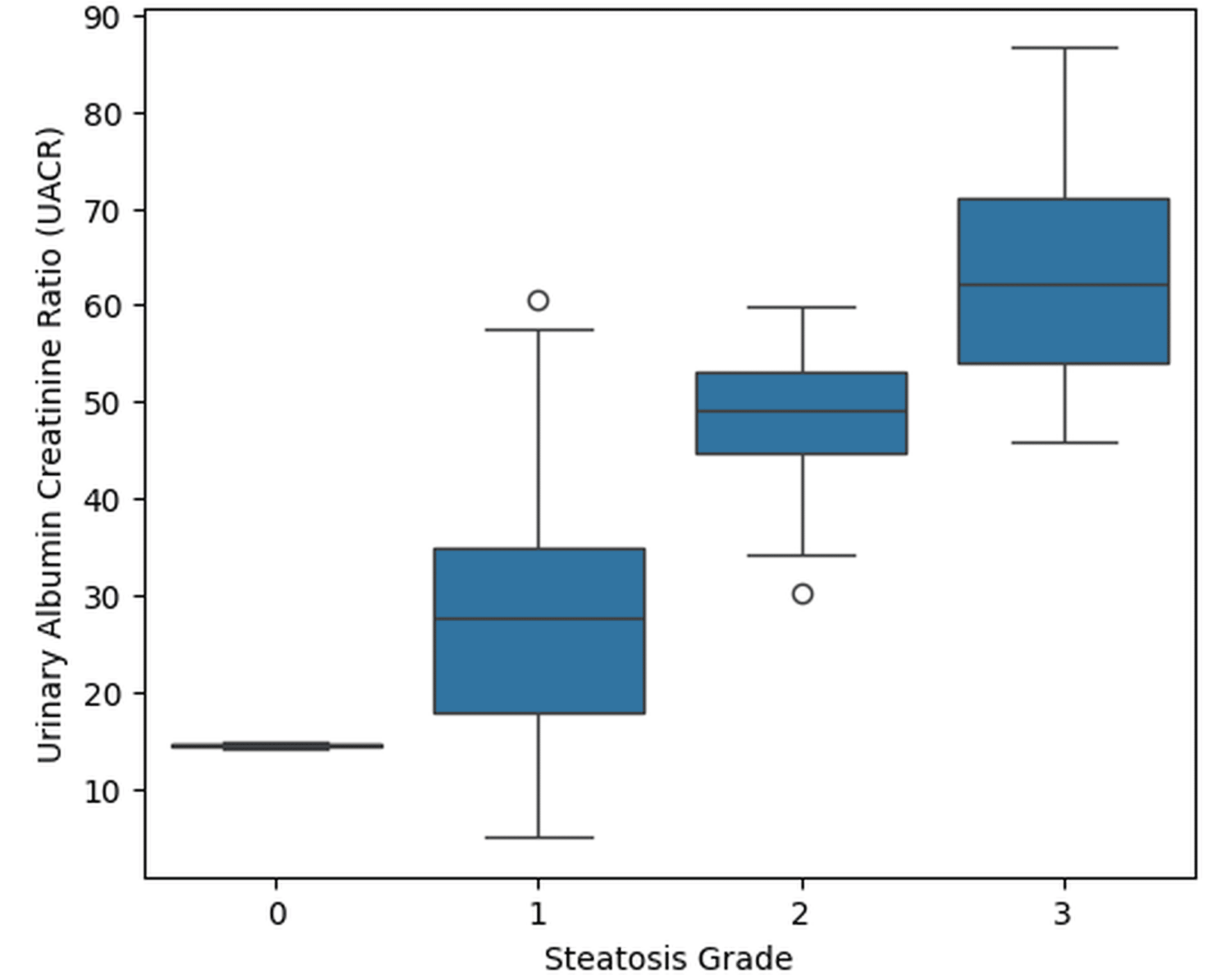
Association between hepatic steatosis grade and urinary albumin–creatinine ratio (UACR). The boxplot demonstrates the distribution of UACR across increasing grades of hepatic steatosis.

The distribution of UACR across different grades of hepatic steatosis is illustrated in Figure 1. Patients without steatosis (grade 0) demonstrated the lowest UACR values, whereas patients with grade 3 steatosis exhibited the highest median UACR levels. Overall, the boxplot demonstrates a stepwise increase in UACR with increasing grades of hepatic steatosis. Comparison of UACR across steatosis grades using the Kruskal-Wallis test demonstrated a statistically significant difference between groups (H = 49.53, p < 0.001).

Correlation between liver attenuation index and UACR

**Figure 2:**
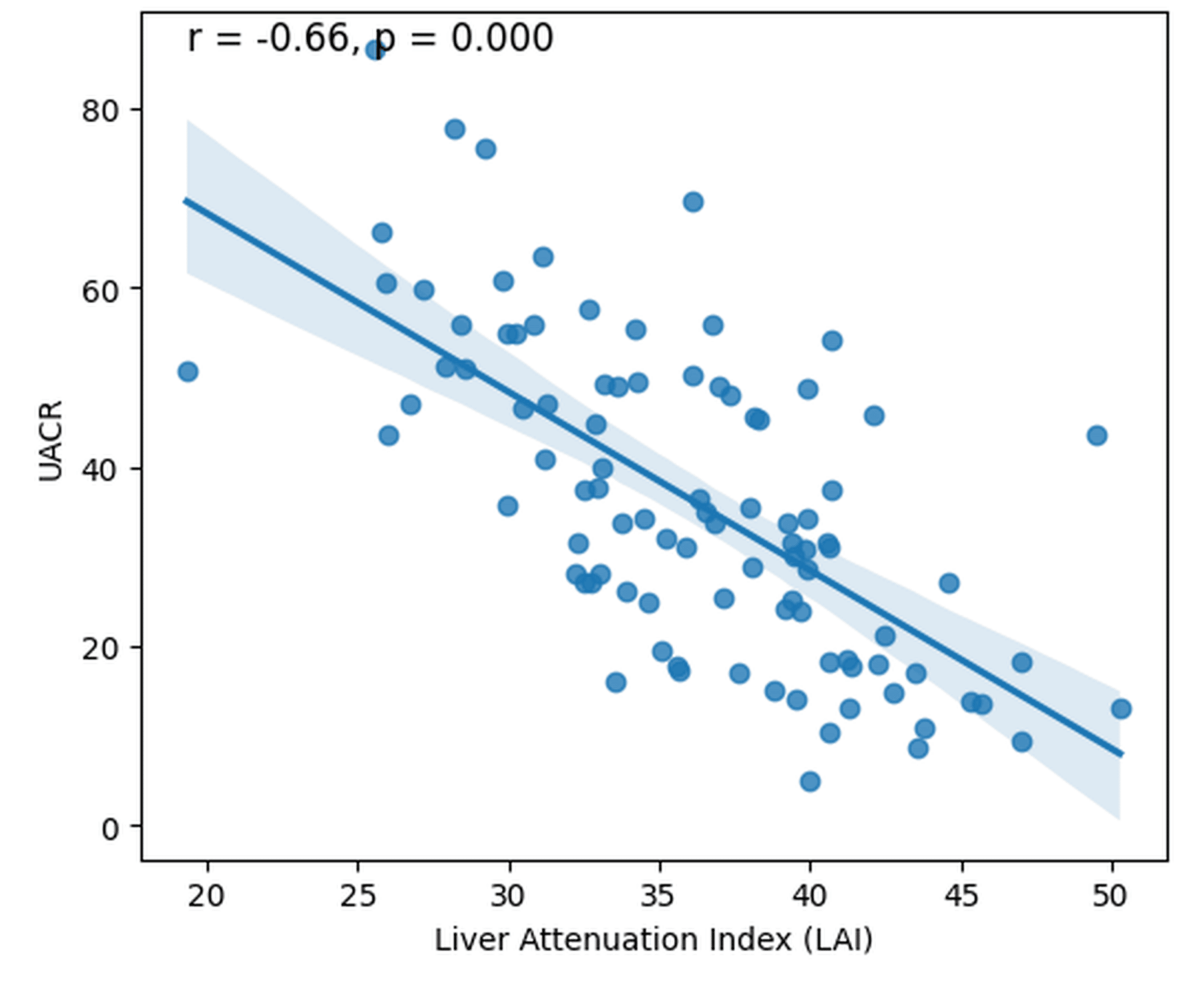
Correlation between liver attenuation index (LAI) and urinary albumin–creatinine ratio (UACR). The scatter plot with fitted regression line demonstrates a significant negative correlation between LAI and UACR (r = −0.66, p < 0.001).

The relationship between LAI and UACR is illustrated in Figure 2. The scatter plot demonstrates a clear inverse linear relationship between LAI and UACR, with lower LAI values associated with higher levels of albuminuria. Pearson correlation analysis revealed a significant negative correlation between LAI and UACR (r = -0.66, p < 0.001), indicating that greater hepatic steatosis burden, reflected by lower LAI values, was associated with increased urinary albumin excretion.

Multivariate regression analysis

Multivariate linear regression analysis was performed to identify independent predictors of UACR, including LAI, BMI, age, HbA1c, and duration of diabetes as independent variables. The overall regression model was statistically significant (F = 17.23, p < 0.001) and explained approximately 49% of the variance in UACR (R² = 0.49). In the adjusted model, LAI demonstrated a strong independent negative association with UACR (β = -1.69, p < 0.001; 95% CI: -2.18 to -1.19). BMI also showed a significant positive association with UACR (β = 2.13, p = 0.004; 95% CI: 0.68 to 3.59). The results of the multivariate regression analysis are summarized in Table 2. Age, HbA1c, and duration of diabetes were not independently associated with UACR.

**Table 2:** Multivariate linear regression analysis for predictors of urinary albumin–creatinine ratio (UACR)

Variable	β Coefficient	Standard Error	t value	p value	95% Confidence Interval
Liver attenuation index (LAI)	−1.69	0.25	−6.76	<0.001	−2.18 to −1.19
Body mass index (BMI)	2.13	0.73	2.92	0.004	0.68 to 3.59
Age	−0.002	0.15	−0.01	0.989	−0.30 to 0.29
Glycated hemoglobin (HbA1c)	0.46	0.58	0.79	0.430	−0.69 to 1.62
Duration of diabetes	0.006	0.20	0.03	0.975	−0.39 to 0.40

## Discussion

The present study evaluated the association between hepatic steatosis burden and microalbuminuria in patients with T2DM. The findings demonstrated a significant relationship between hepatic steatosis and urinary albumin excretion. Specifically, LAI and L/S ratio, a CT imaging marker of hepatic fat accumulation, showed a strong inverse correlation with UACR. In addition, increasing grades of hepatic steatosis were associated with progressively higher levels of albuminuria. Multivariate regression analysis further demonstrated that LAI and BMI were independently associated with UACR, suggesting that hepatic steatosis and adiposity may contribute to renal endothelial dysfunction in individuals with T2DM.

The findings of the present study are consistent with previous evidence demonstrating an association between MASLD and chronic kidney disease. MASLD is increasingly recognized as a multisystem metabolic disorder affecting not only the liver but also the cardiovascular and renal systems. Byrne and Targher described MASLD as a condition closely linked to insulin resistance, obesity, and cardiometabolic complications [3]. Similarly, Targher et al. demonstrated that patients with NAFLD had a significantly higher prevalence of chronic kidney disease compared with individuals without hepatic steatosis, independent of traditional metabolic risk factors [4].

Several meta-analyses and large observational studies have further supported this association [6-8,10,11]. Musso et al. demonstrated that NAFLD was associated with an increased risk of incident chronic kidney disease [6], while Mantovani et al. reported similar findings in individuals with T2DM [7]. Recent population-based studies have also shown that MASLD is independently associated with increased risk of chronic kidney disease and albuminuria [8,10,11]. The findings of the present study are in agreement with these observations and suggest that hepatic steatosis burden may be closely linked to early renal endothelial dysfunction.

The inverse relationship observed between LAI and UACR suggests that greater hepatic fat accumulation is associated with worsening albuminuria. Several pathophysiological mechanisms may explain this association. MASLD and diabetic kidney disease share common metabolic abnormalities, including insulin resistance, oxidative stress, systemic inflammation, and endothelial dysfunction [3]. Excess hepatic fat accumulation promotes the release of inflammatory cytokines, adipokines, and hepatokines that contribute to systemic metabolic dysregulation [12]. These mediators may promote glomerular endothelial injury and increased glomerular permeability, ultimately resulting in albuminuria.

Adiposity also appeared to play an important role in the present study, as BMI demonstrated an independent positive association with UACR. Obesity is known to exacerbate insulin resistance and chronic low-grade inflammation, both of which contribute to the development of microvascular complications in T2DM. Stefan et al. highlighted the important role of ectopic fat deposition and adipose tissue dysfunction in the pathogenesis of metabolic complications associated with fatty liver disease [13]. Altered adipokine secretion, including adiponectin and leptin dysregulation, has also been implicated in both hepatic steatosis and renal injury [14].

The progressive increase in UACR across increasing grades of hepatic steatosis observed in the present study further supports the relationship between hepatic fat accumulation and renal dysfunction. Patients with higher grades of steatosis demonstrated higher median UACR levels and greater variability in albuminuria. These findings suggest that increasing hepatic fat burden may reflect a more severe metabolic phenotype associated with microvascular complications.

The clinical implications of these findings are noteworthy. Hepatic steatosis is increasingly recognized as a marker of systemic metabolic dysfunction. Identification of hepatic steatosis in patients with T2DM may help identify individuals at increased risk of renal endothelial dysfunction and early diabetic kidney disease. Imaging-based parameters such as LAI may therefore provide additional insights into cardiometabolic risk stratification in individuals with T2DM. However, due to the cross-sectional design, these findings should be interpreted as associative rather than causal, and prospective longitudinal studies are needed to determine temporal relationships and causality.

Recent evidence has also suggested that hepatic fat accumulation may contribute to renal injury through hepatokine-mediated mechanisms, including altered secretion of fibroblast growth factor-21 and fetuin-A, both of which influence insulin resistance and endothelial dysfunction [5,15,16]. These observations further support the biological plausibility of the association between hepatic steatosis and microalbuminuria demonstrated in the present study.

Interestingly, glycemic control as reflected by HbA1c did not demonstrate an independent association with UACR in the present study, although a weak positive correlation was observed. This finding suggests that hepatic steatosis and underlying metabolic dysfunction may contribute to albuminuria through mechanisms beyond hyperglycemia alone. In addition, genetic susceptibility and variability in long-term glycemic exposure may partly explain interindividual differences in renal dysfunction among patients with similar glycemic status. Furthermore, the relatively modest sample size and presence of outliers may have reduced the statistical power to detect a significant association.

The present study has several strengths. Hepatic steatosis was assessed using CT-derived quantitative parameters, including LAI and steatosis grading, allowing objective evaluation of hepatic fat burden. In addition, both correlation analysis and multivariate regression modeling were used to evaluate the relationship between hepatic steatosis and albuminuria. The study population also consisted exclusively of patients with T2DM, a group at particularly high risk for both hepatic steatosis and renal complications.

However, certain limitations should be acknowledged. The cross-sectional design limits the ability to establish causality between hepatic steatosis and albuminuria. The study was conducted at a single tertiary care center with a relatively modest sample size, which may limit the generalizability of the findings. In addition, hepatic steatosis was assessed using imaging parameters rather than histopathological evaluation, which is the gold standard. Further longitudinal studies with larger cohorts are required to better clarify the causal relationship between hepatic steatosis and diabetic kidney disease. 

Additional potential confounding factors, including antihypertensive treatment (particularly renin-angiotensin system blockers), lipid-lowering medications, smoking status, and duration/severity of hypertension, were not systematically evaluated and may have influenced albuminuria outcomes. Furthermore, the relatively high HbA1c values observed in the cohort indicate suboptimal glycemic control, which itself may contribute to renal dysfunction. 

## Conclusions

The present study demonstrated a significant association between hepatic steatosis burden and microalbuminuria in patients with T2DM. Lower LAI values and higher grades of hepatic steatosis were associated with increased urinary albumin excretion independent of several metabolic variables. These findings suggest that hepatic steatosis may represent an important marker of early renal endothelial dysfunction in individuals with T2DM.

Recognition of hepatic steatosis in patients with T2DM may therefore aid in identifying individuals at increased risk of diabetic kidney disease and may have implications for early risk stratification and integrated metabolic management. Further longitudinal studies with larger sample sizes are required to clarify the causal relationship between hepatic steatosis and renal dysfunction.
